# Patterns of flea infestation in rodents and insectivores from intensified agro-ecosystems, Northwest Spain

**DOI:** 10.1186/s13071-020-04492-6

**Published:** 2021-01-06

**Authors:** Silvia Herrero-Cófreces, Manuel Fabio Flechoso, Ruth Rodríguez-Pastor, Juan José Luque-Larena, François Mougeot

**Affiliations:** 1 Dpto. Ciencias Agroforestales, ETSIIAA, Universidad de Valladolid, Avda. de Madrid 44, 34004 Palencia, Spain; 2 Instituto Universitario de Investigación en Gestión Forestal Sostenible, Palencia, Spain; 3 Dpto. Biología Animal (Zoología), Universidad de Salamanca, Campus Unamuno S/N, 37007 Salamanca, Spain; 4 Instituto de Investigación en Recursos Cinegéticos, IREC (CSIC-UCLM-JCCM), Ronda de Toledo s/n, 13071 Ciudad Real, Spain

**Keywords:** Aggregation, *Apodemus sylvaticus*, *Crocidura russula*, Ectoparasite, Host sex effects, *Microtus arvalis*, *Mus spretus*, Seasonal variations, Siphonaptera co-infection

## Abstract

**Background:**

Fleas frequently infest small mammals and play important vectoring roles in the epidemiology of (re)emerging zoonotic disease. Rodent outbreaks in intensified agro-ecosystems of North-West Spain have been recently linked to periodic zoonotic diseases spillover to local human populations. Obtaining qualitative and quantitative information about the composition and structure of the whole flea and small mammal host coexisting communities is paramount to understand disease transmission cycles and to elucidate the disease-vectoring role of flea species. The aims of this research were to: (i) characterise and quantify the flea community parasiting a small mammal guild in intensive farmlands in North-West Spain; (ii) determine and evaluate patterns of co-infection and the variables that may influence parasitological parameters.

**Methods:**

We conducted a large-scale survey stratified by season and habitat of fleas parasitizing the small mammal host guild. We report on the prevalence, mean intensity, and mean abundance of flea species parasitizing *Microtus arvalis*, *Apodemus sylvaticus*, *Mus spretus* and *Crocidura russula*. We also report on aggregation patterns (variance-to-mean ratio and discrepancy index) and co-infection of hosts by different flea species (Fager index) and used generalized linear mixed models to study flea parameter variation according to season, habitat and host sex.

**Results:**

Three flea species dominated the system: *Ctenophthalmus apertus gilcolladoi, Leptopsylla taschenbergi* and *Nosopsyllus fasciatus*. Results showed a high aggregation pattern of fleas in all hosts. All host species in the guild shared *C. a. gilcolladoi* and *N. fasciatus*, but *L. taschenbergi* mainly parasitized mice (*M. spretus* and *A. sylvaticus*). We found significant male-biased infestation patterns in mice, seasonal variations in flea abundances for all rodent hosts (*M. arvalis, M. spretus* and *A. sylvaticus*), and relatively lower infestation values for voles inhabiting alfalfas. Simultaneous co-infections occurred in a third of all hosts, and *N. fasciatus* was the most common flea co-infecting small mammal hosts.

**Conclusions:**

The generalist *N. fasciatus* and *C. a. gilcolladoi* dominated the flea community, and a high percentage of co-infections with both species occurred within the small mammal guild. *Nosopsyllus fasciatus* may show higher competence of inter-specific transmission, and future research should unravel its role in the circulation of rodent-borne zoonoses.
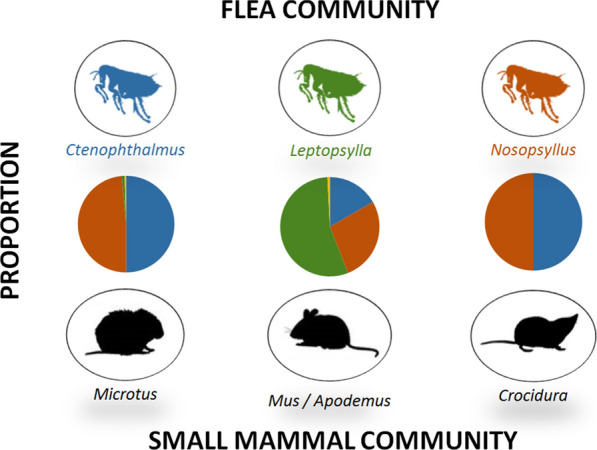

## Background

Fleas are abundant ectoparasites that frequently infest mammals and birds [1]. These haematophagous insects can act as vectors of numerous pathogens transmitted through biting or by direct contact with their faeces [1] and often play a relevant role in the circulation and epidemiology of emerging and re-emerging diseases worldwide [1, 2]. Fleas spread the well-studied plague, caused by *Yersinia pestis* [3], as well as other emerging pathogens that cause zoonoses such as rickettsioses (murine typhus and flea-borne spotted fever) and bartonelloses [1]. Fleas also maintain and transmit pathogens causing tularemia, Q fever, trypanosomiasis and myxomatosis and can act as intermediate hosts for some helminthiases [1, 4]. Comprehensive knowledge of flea ecology becomes essential to uncover their role in the circulation of diseases in nature. For instance, patterns of flea distribution and abundance in host communities, seasonal variation of such patterns, relationships with individual host characteristics (e.g., host sex, age, condition, immune function), the effect of environmental habitat and conditions (temperature, humidity) and co-infection, are all relevant aspects that need to be quantified to build any baseline knowledge required to understand flea life cycles and their relative ecological and epidemiological roles [5, 6].

Many fleas parasitize rodents [1], which account for 25% of all living mammals and act as the main host type for > 80% of all known flea species [5]. Rodents are a key mammal group in terms of public health, as they are involved in the amplification and spillover of many zoonoses affecting humans globally [7], and their fleas often play significant vectoring roles in the transmission cycles of disease [7–9]. In Northwest Spain, common vole (*Microtus arvalis*) populations massively invaded lowland agricultural landscapes between the 1970s and 1990s, putatively colonizing newly irrigated fodder crops from natural peripheral mountainous habitats [10–12]. In recently colonized farmland, common vole populations are cyclic [13] and periodically become a crop pest when overabundant, causing serious public health impacts due to the amplification and spillover of zoonotic diseases like tularemia [10, 14, 15]. In these intensively farmed landscapes, common voles coexist in the same microhabitats with other sympatric rodents and insectivores (mainly, wood mouse *Apodemus sylvaticus*, Algerian mouse *Mus spretus* and greater white-toothed shrew *Crocidura russula*) [16]. Characteristics of the host (morphological, physiological, immunological, behavioural and phylogenetic traits) and their shelters (i.e. burrows, nests) are critical to flea lifecycles [1, 5]. In other study systems, flea specificity is an important trait influencing the flea community [17, 18]. Fleas can infest hosts phylogenetically close [18], switching between coexisting species within guilds [19]. Host density is also a relevant factor to consider since it involves variations in flea species richness [20, 21]. Furthermore, climatic conditions, local factors and specific host features shape infestation patterns at the local level [18, 22–24]. Due to all these sources of variation, general patterns should not be inferred but studied in detail in local flea communities.

In the Palearctic region, up to 6 different taxonomic families of fleas can infest small mammals [1], and 68 flea species have been identified to date in the Iberian peninsula [25]. Three of these flea families occur in Northwest Spain: Ceratophyllidae, Ctenophthalmidae and Pulicidae [26–29]. Recent surveys reported that the main flea species infesting common voles in intensive farmland of Northwest Spain were *Ctenophthalmus apertus*, *Nosopsyllus fasciatus* and *Leptopsylla taschenbergi* [30].

Fleas parasitizing common voles in Northwest Spain are known to harbour zoonotic bacteria such as *Francisella tularensis* (i.e. the etiological agent of tularemia) and several *Bartonella* species (agent of bartonelloses) [30]. Yet, nothing is known about flea distribution, relative abundance and co-infection patterns within and between the small mammal host guild. Improving our basic knowledge on how fleas interact with their local hosts in farming landscapes will aid in the understanding of disease circulation in landscapes frequently scourged by rodent-driven zoonoses. Here we report on the patterns of flea infestation in the small mammal community inhabiting the intensively farmed landscapes in Northwest Spain dominated by colonizing common voles. Specifically, we document and quantify flea-host specificity and describe patterns of abundance, prevalence, intensity and aggregation of each flea species on each of the main small mammal hosts. We also evaluated patterns of flea co-infection in hosts and studied relative abundance variation according to season, habitat (i.e. crop type) and host sex.

## Methods

### Study area

The study was conducted in intensively farmed landscapes in the Castilla-y-León region, Northwest Spain. These landscapes are steppe-like crop mosaics dominated by cereal fields (mainly wheat and barley) with scattered irrigated crops (e.g. sweet beet, sunflower, corn and alfalfa) and interspersed by remnant semi-natural vegetation (fallows or set-aside, field margins, grassy road verges and wildflower strips; [16]). Climate is continental-Mediterranean, characterized by wide seasonal temperature oscillations: long, cold and humid winters with frequent frost events, followed by dry and hot summers with variable but persistent drought periods; precipitation is mostly concentrated during spring and autumn [31].

### Study small mammals

In the studied habitats, the bulk (> 95%) of the small mammal community includes three rodents and one insectivore: common voles (*M. arvalis*), wood mice (*A. sylvaticus*), Algerian mice (*M. spretus*) and white-toothed shrews (*C. russula*) [16]. The common vole is a fossorial rodent [32] characterized by population peaks recorded every 3 years [13], whereas the two mouse species show seasonal fluctuations [33, 34]. *Microtus arvalis* have a preference for permanent herbaceous fields [16], while *A. sylvaticus* is a habitat generalist species [33] and *M. spretus* prefers Mediterranean open habitat ecosystems [34]. The white-toothed shrew selects forest edges and open habitats with high vegetation cover [35].

### Small mammal trappings

Fieldwork consisted of seasonal live trappings (March, July and November) conducted at three independent localities (> 60 km apart) in the provinces of Palencia (42°01ʹN, 4°42ʹW), Valladolid (41°34ʹN, 5°14ʹW) and Zamora (41°50ʹN, 5°36ʹW), all within the Castilla-y-León region. Between July 2009 and July 2015, we monitored six study areas (two replicates per locality, each replicate consisted of an area of *c.* 40 km^2^). In each study area, we sampled the three most relevant habitats: cereals (most abundant crop type), alfalfas (most favourable crop type in terms of cover and food availability for voles) and fallows (see [16] for more details on trapping procedures and habitat use by voles). In brief, we randomly selected 12 fields (4 cereals, 4 alfalfas and 4 fallows) amongst those available in a given area, avoiding sampling the same locations during consecutive seasonal trappings, and set 35 traps in each field (8 cm × 9 cm × 23 cm; LFAHD Sherman©) interspaced 2 m and deployed in a T-line shape from field margins towards the inside of crop fields [16]. Traps were baited with carrot and apple and set for 24 h. Each trapped animal was provided with a unique code (we noted the date, site and crop field where it was trapped). For this study, we captured and sampled 2254 small mammals belonging to the species *M. arvalis* (61.2%), *A. sylvaticus* (23.1%), *M. spretus* (13.5%), *C. russula* (1.9%) and other species (0.3%).

### Flea collection from trapped animals and identification

Captured rodents and shrews were individually transferred to laboratory animal plastic cages (29 × 22 × 14 cm; Panlab®). To follow the ethical legislation about the welfare of animals used in research [36], and to maximise the number of animals that arrived alive to the laboratory, we provided captured animals with food, water and bedding material until the euthanasia procedure. In the laboratory, each animal was sexed, weighted and killed with CO_2_, following a humane protocol approved by the University of Valladolid Ethics Committee for Animal Research (code: 4801646). Immediately after death, fleas were carefully collected from each animal by blowing the fur and combing it with a lice comb while holding the animal over a white plastic pan (520 × 420 × 95 mm) half-filled with water. Fleas from each individual were counted, collected from the water surface using a pair of tweezers and stored in individually labelled tubes filled with 70% ethanol [37, 38]. We ensured that no fleas were missed from each individual by placing the animal carcasses in sealed plastic bags and leaving them for 1 h in the refrigerator before checking again for fleas. Fleas were subsequently studied with a 10× and 40× optical microscope (Nikon Optiphot-2) and identified at the species level using dichotomous keys [39]. We collected a total of 4715 fleas from 1239 small mammal hosts: 3900 fleas from *M. arvalis* (n = 941), 698 from *A. sylvaticus* (n = 238), 87 from *M. spretus* (n = 49), 14 from *C. russula* (n = 6) and 16 from other small mammals (n = 5). A total of 4266 individual fleas were identified.

### Data analysis

For each host species, we obtained information on the prevalence, mean abundance and mean intensity of each flea species (following Bush et al. [40]). Data were summarized as prevalence ± 95% confidence intervals (CI; traditional Clopper-Pearson confidence limits) and mean intensity or abundance ± standard error (SE). We also quantified the level of skewness of the flea distribution on hosts (a measure of the asymmetry of the probability distribution of a real-valued random variable about its mean) using two complementary indices: (i) the variance-to-mean ratio (VMR); (ii) the discrepancy index (*D*) following Poulin [41]. Co-infection was also quantified (hosts infested by more than one flea species). These descriptive statistics were obtained using the Quantitative Parasitology (QPweb) software version 1.0.14 [42]. We used the Fager index [43] to determine the degree of co-occurrence of flea species, regardless of abundance variations. This index ranges between 0 (species never infest simultaneously) and 1 (species always co-occur) and was calculated as follows:

$$I_{{{\text{AB}}}} = 2J/(N_{{\text{A}}} + N_{{\text{B}}})$$

*J* is the number of hosts where parasitic species A and B are present simultaneously; *N*_A_ is the number of hosts with species A present; *N*_B_ is the number of hosts with species B present.

Co-infection differences according to host sex were tested using Pearson’s chi-square tests or G-tests, depending on minimum sample sizes. For rodent hosts, we studied flea prevalence, mean intensity and mean abundance variation according to sampling month, crop type (except for *M. spretus* because of the small sample size) and host sex. We used generalized linear mixed-effects models (GLMM) and a negative binomial distribution for intensity and abundance. Models included the factors year (2009–2015) and site (Palencia/Valladolid/Zamora) as random terms (to account for possible temporal or spatial variations) whenever possible and the factors habitat (crop), type (alfalfa/cereal/fallow), host sex (male/female), and season/month (November/March/July) as explanatory variables. Because of sample size limitations, some mixed models did not converge. We then included site or site and year as fixed effects instead of random effects. The model selection followed a backwards selection procedure (using the “drop1” function in R), sequentially removing non-significant interactions and factors (we report both significant, *P* = 0.05 level, and marginally significant, *P* = 0.10 level, effects). Differences between levels of the categorical factors (crop type and month) were tested using *post-hoc* Tukey tests. Statistical models were carried out using the “lme4” [44] and “R2admb” [45] packages, and G-tests using “RVAideMemoire” [46] of the R3.6.1 software [47].

## Results

### Flea community

**Fig. 1:**
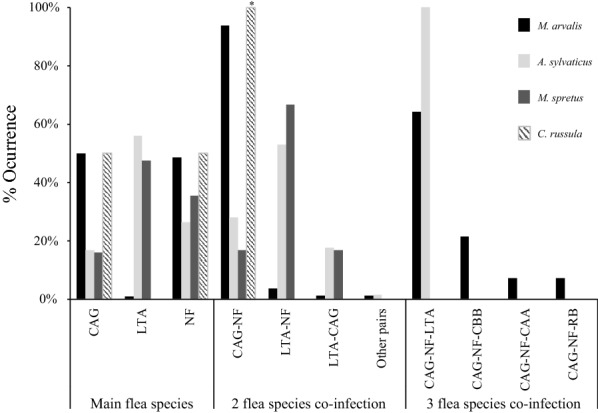
Flea abundance frequencies in the main small mammal host species (Rodents and Insectivores). CAA, *Ctenophthalmus apertus apertus*; CAG, *Ctenophthalmus apertus gilcolladoi*; CB, *Ctenophthalmus baeticus*; NF, *Nosopsyllus fasciatus*; LT, *Leptopsylla taschenbergi*; RB, *Rhadinopsylla beillardae*; * Sample size too small (*n* = 1)

The flea community included the following species: *Ctenophthalmus apertus apertus* (*n* = 2), *C. a. gilcolladoi* (*n* = 1879), *C. baeticus baeticus* (*n* = 11), *Leptopsylla taschenbergi amitina* (*n* = 460), *Nosopsyllus fasciatus* (*n* = 1903) and *Rhadinopsylla beillardae* (*n* = 8); in addition, two specimens were identified at genus level only (*Ctenophthalmus* spp*.*). Two species dominated the small mammal flea community: *N. fasciatus* and *C. a. gilcolladoi* (frequency = 44.6% and 44.1%, respectively), followed by *L. taschenbergi* (10.8%). Patterns of flea infestation differed between small mammal host species (Table 1 and Fig. 1; complementary information in Additional file 1: Figure S1). The most abundant flea species infesting common voles were *N. fasciatus* and *C. a. gilcolladoi,* representing 98.5% of all fleas identified (48.5% and 50.0%, respectively). By contrast, *L. taschenbergi* was the most abundant flea infesting mice (56% and 48% of all fleas for *A. sylvaticus* and *M. spretus*, respectively). Shrews were only infested by *C. a. gilcolladoi* and *N. fasciatus*. Other *Ctenophthalmus* spp. different from *C. a. gilcolladoi* (*C. a. apertus*, *C. baeticus* and *Ctenophthalmus* spp.) were seldom found in *M. arvalis* (in one, eight and two animals, respectively). The flea *R. beillardae* was occasionally identified in the most abundant rodent species (two fleas in two *M. arvalis*, five fleas in two *A. sylvaticus* and one flea in one *M. spretus*).

**Table 1.** Parasitological parameters of the flea community of studied small mammal hosts studied

Host species [*n* total^a^/*n* alive^b^]	Flea species	*n*. identified fleas [n hosts^c^]	Fleas intensity range	Prevalence % (95% CI)^d^	Mean intensity (± SE)	Mean abundance (± SE)	Variance/mean ratio	Discrepancy index Mean (95% CI)^**e**^
*Microtus arvalis* [1380/941]	CAG	1731 [539]	1–18	39.1 (36.5-41.7)	3.21 (0.14)	1.25 (0.09)	9.8	0.81^f^ (0.79-0.83)
	NF	1681 [643]	1–30	46.6 (43.9-49.3)	2.61 (0.09)	1.22 (0.06)	4.19	0.73^f^ (0.71-0.75)
	LT	34 [29]	1–4	2.1 (1.4-3.0)	1.17 (0.01)	0.02 (0.01)	1.45	0.98^f^ (0.97-0.99)
*Apodemus sylvaticus* [522/238]	CAG	116 [75]	1–7	14.4 (11.5-17.7)	1.55 (0.06)	0.22 (0.03)	2.2	0.90 (0.87-0.92)
	NF	182 [116]	1–7	22.2 (18.7-26.0)	1.57 (0.07)	0.35 (0.04)	1.97	0.84 (0.81-0.87)
	LT	387 [140]	1–23	26.8 (23.1-30.8)	2.76 (0.17)	0.74 (0.09)	5.21	0.85 (0.82-0.87)
*Mus spretus* [304/49]	CAG	13 [11]	1–3	3.6 (1.8-6.4)	1.18 (0.09)	0.04 (0.01)	1.42	0.97 (0.95-0.98)
	NF	29 [21]	1–5	6.9 (4.3-10.4)	1.38 (0.14)	0.10 (0.02)	1.88	0.94 (0.92-0.97)
	LT	39 [18]	1–7	5.9 (3.5-9.2)	2.17 (0.22)	0.13 (0.04)	3.24	0.96 (0.94-0.97)
*Crocidura russula* [42/6]	CAG	7 [2]	2–5	4.8 (0.6-16.2)	3.50 (0.83)	0.17 (0.13)	4.07	0.94 (0.86-0.95)
	NF	7 [5]	1–3	11.9 (4.0-25.6)	1.40 (0.40)	0.17 (0.08)	1.73	0.89 (0.79-0.95)
*Microtus lusitanicus/duodecimcostatus* [4/3]	CAG	12 [3]	2–6	–	–	–	–	–
	NF	2 [1]	2	–	–	–	–	–
*Mustela nivalis* [2/2]	NF	2 [2]	1	–	–	–	–	–

SE, standard error; CAG, *Ctenophthalmus apertus gilcolladoi*; NF, *Nosopsyllus fasciatus*; LT, *Leptopsylla taschenbergi*.

^a^Number of total hosts captured

^b^Number of hosts brought alive to the laboratory and killed, infested or uninfested

^c^Number of hosts infested with all fleas identified

^d^95% Confidence interval by Clopper-Pearson

^e^95% Confidence interval by bootstrap method

^f^Sample too big for bootstrap confident limits; the percentile method was used instead

Overall, flea prevalence on small mammal hosts averaged 51.6% (CI = 49.5–53.7), and intensity averaged 3.66 fleas per infested host (SE = ± 0.15; range = 0–68), resulting in a mean abundance of 1.89 (SE = ± 0.09). Detailed information on prevalence, mean abundance and mean intensity of each flea species and host is provided in Table 1. Fleas were highly aggregated on their small mammal hosts (Table 1; D-index values close to 1). Variance-to-mean ratios were also indicative of a marked parasite aggregation, with greater ratios for those fleas typically more abundant on a given host (*C. a. gilcolladoi* and *N. fasciatus* for *M. arvalis*; *L. taschenbergi* for *A. sylvaticus* and *M. spretus*).

### Variation of flea parasitological parameters according to season, crop type and host sex

Flea prevalence, mean intensity and mean abundance were highest in *M. arvalis* (68.2%, CI = 65.7–70.6; 4.14, SE = ± 0.19; and 2.83, SE = ± 0.13, respectively) and *A. sylvaticus* (45.6%, CI = 413–50.0; 2.93, SE = ± 0.20; and 1.34, SE = ± 0.11, respectively) and noticeably lower in *M. spretus* (16.1%, CI = 12.2–20.7; 1.78, SE = ± 0.21; and 0.29, SE = ± 0.48, respectively) and *C. russula* (14.3%, CI = 5.40–28.5; 2.33, SE = ± 0.67; and 0.33, SE = ± 0.15, respectively). The most fleas per host were harboured by *M. arvalis* [range 1–68], followed by *A. sylvaticus* [range 1–29], while *M. spretus* [range 1–5] and *C. russula* [range 1–7] had fewer fleas per host. For further analyses of infestation patterns, we focused on the main flea species (*C. a. gilcolladoi*, *N. fasciatus* and *L. taschenbergi*) and the most frequently captured small mammal hosts (*M. arvalis*, *A. sylvaticus* and *M. spretus*) (Table 2).

**Table 2:** Results of GLMMs (best models) explaining flea parameters of main rodent host species

Host	Fleas	Prevalence			Mean abundance			Mean intensity		
		Predictor	Estimate ± SE	*Z*-value	Predictor	Estimate ± SE	Z-value	Predictor	Estimate ± SE	*Z*-value
*M. arvalis*	All	Crop (cereal)	0.40 ± 0.16	2.57*	Crop (cereal)	0.51 ± 0.09	5.37***	Crop (cereal) ^a^	0.38 ± 0.73	5.16***
		Crop (fallow)	0.38 ± 0.14	2.67**	Crop (fallow)	0.47 ± 0.09	5.43***	Crop (fallow)	0.36 ± 0.66	5.47***
		Month (July)	0.60 ± 0.09	3.17**	Month (July)	− 0.20 ± 0.12	− 1.64	Month (July)	− 0.31 ± 0.10	− 3.16**
		Month (November)	0.47 ± 0.23	2.02*	Month (November)	0.04 ± 0.15	0.29	Month (November)	− 0.13 ± 0.12	− 1.11
	CAG	Crop (cereal)	0.24 ± 0.15	1.62	Crop (cereal)	0.30 ± 0.13	2.29*	Crop (cereal)	0.19 ± 0.12	1.56
		Crop (fallow)	2.29 ± 0.14	2.16*	Crop (fallow)	0.45 ± 0.12	3.77***	Crop (fallow)	0.34 ± 0.12	3.14**
		Month (July)	− 0.51 ± 0.19	− 2.74**	Month (July)	− 0.66 ± 0.16	− 3.98***	Month (July)	− 0.81 ± 0.15	− 5.23***
		Month (November)	0.31 ± 0.23	1.34	Month (November)	0.05 ± 0.19	0.28	Month (November)	− 0.15 ± 0.18	− 0.81
	NF	Crop (cereal)	0.62 ± 0.15	4.23***	Crop (cereal)	0.68 ± 0.11	6.24***	Crop (cereal)	0.50 ± 0.09	5.35***
		Crop (fallow)	0.55 ± 0.13	4.09***	Crop (fallow)	0.49 ± 0.10	4.82***	Crop (fallow)	0.34 ± 0.09	3.86***
		Month (July)	0.88 ± 0.20	4.46***	Month (July)	0.68 ± 0.11	6.24**	Month (July)	0.26 ± 0.14	1.92^
		Month (November)	0.35 ± 0.24	1.44	Month (November)	0.40 ± 0.15	0.54	Month (November)	− 0.13 ± 0.16	− 0.78
	LT	Sex (male)	0.72 ± 0.39	1.86^	Sex (male)	0.87 ± 0.41	2.12*	Sex (male)	0.90 ± 0.41	2.14*
*A. sylvaticus*	All	Month (July)	1.33 ± 0.29	4.54***	Month (July)^a^	1.01 ± 0.20	5.05***	Month (July) ^a^	0.39 ± 0.16	2.47*
		Month (November)	− 0.08 ± 0.31	− 0.27	Month (November)	− 0.40 ± 0.24	− 1.68^	Month (November)	− 0.28 ± 0.21	− 1.33
		Sex (male)	0.62 ± 0.21	2.94**	Sex (male)	0.50 ± 0.34	3.42***	Sex (male)	0.27 ± 0.11	2.35*
	CAG	Sex (male)	0.76 ± 0.30	2.54*	Sex (male)	0.87 ± 0.52	3.07**	Month (July)	− 1.03 ± 0.29	− 3.54***
								Month (November)	− 0.64 ± 0.39	− 1.66^
								Sex (male)	0.77 ± 0.27	2.85**
	NF	Month (July) ^a^	0.93 ± 0.31	2.95**	Month (July)^a^	0.52 ± 0.26	2.03*	Month (July)^a^	− 0.20 ± 0.23	− 0.87
		Month (November)	− 0.24 ± 0.36	− 0.64	Month (November)	− 0.65 ± 0.33	− 1.99*	Month (November)	− 0.71 ± 0.30	− 2.35*
	LT	Month (July)	2.40 ± 0.42	5.64***	Crop (cereal)^a^^,^^b^	0.50 ± 0.22	2.27*	Crop (cereal)^a^^,^^b^	0.31 ± 0.19	1.63
		Month (November)	0.45 ± 0.47	0.97	Crop (fallow)	− 0.16 ± 0.25	− 0.63	Crop (fallow)	− 0.24 ± 0.23	− 1.07
		Sex (male)	1.02 ± 0.25	4.08***	Month (July)	2.56 ± 0.40	6.44***	Month (July)	1.95 ± 0.38	5.09***
					Month (November)	0.40 ± 0.44	0.90***	Month (November)	0.46 ± 0.45	1.01
					Sex (male)	0.86 ± 0.21	4.20	Sex (male)	0.50 ± 0.18	2.72**
*M. spretus*	All	Month (July) ^a^	1.38 ± 0.59	2.33*	Month (July)^a^^,^^b^	1.33 ± 0.67	1.99*	NA	–	–
		Month (November)	0.23 ± 0.52	0.43	Month (November)	0.18 ± 0.62	0.29			
		Sex (male)	0.98 ± 0.39	2.50*	Sex (male)	1.21 ± 0.41	2.93**			
	CAG	NONE	–	–	NA	–	–	NA	–	–
	NF	Month (July) ^a^	2.37 ± 1.13	2.01*	Month (July)^a^	2.27 ± 1.13	2.01*	NA	–	–
		Month (November)	1.43 ± 1.06	1.35	Month (November)	1.81 ± 1.08	1.67^			
					Sex (male)	0.93 ± 0.58	1.61			
	LT	Month (July)^a^^,^^b^	0.73 ± 1.34	0.55	Month (July)^a^^,^^b^	0.48 ± 1.44	0.33	Sex (male)^a^	1.07 ± 0.68	1.58
		Month (November)	− 1.01 ± 1.35	− 0.75	Month (November)	− 1.48 ± 1.44	− 1.03			
		Sex (male)	1.28 ± 0.68	1.88^	Sex (male)	1.60 ± 0.73	2.20*			

For flea species abbreviation (CAG, LT, NF) see Fig. 1

*NA* model did not converge, *NONE* no significant variables, *SE* standard error

^a^Province not included as random effect

^b^GLM instead of GLMM

^***^p < 0.001; **< 0.01; *< 0.05; ^ marginally significant [0.05–0.10]

Variation in *C. a. gilcolladoi* parameters on *M. arvalis* were significantly explained by month (prevalence: *Χ*^*2*^ = 26.82, *df* = 2, *P* < 0.001; intensity: *Χ*^*2*^ = 46.35, *df* = 2, *P* < 0.001; abundance: *Χ*^*2*^ = 34.53, *df* = 2, *P* < 0.001) and crop type (prevalence: *Χ*^*2*^ = 5.25, *df* = 2, *P* = 0.073*; intensity: *Χ*^*2*^ = 9.85, *df* = 2, *P* = 0.007; abundance: *Χ*^*2*^ = 14.65, *df* = 2, *P* < 0.001), but not by host sex. *Post-hoc* tests (Tukey) indicated that *C. a. gilcolladoi* infestation was less frequent and severe on voles in July than March, with intermediate values in November. Moreover, voles caught in alfalfa had lower flea prevalence, abundance and intensity than those from fallows. In *A. sylvaticus, C. a. gilcolladoi* parameters differed between sexes (prevalence: *Χ*^*2*^ = 6.45, *df* = 1, *P* = 0.011; intensity: *Χ*^*2*^ = 8.10, *df* = 1, *P* = 0.004; abundance: *Χ*^*2*^ = 9.44, *df* = 1, *P* = 0.002), being greater for male than female hosts. The only exception was mean intensity, which reached higher values in animals trapped during March than in July. In *M. spretus,* neither variable explained *C. a. gilcolladoi* prevalence variation. The small sample size for this species did not allows us to analyse intensity or abundance.

Regarding *N. fasciatus*, we found in voles the same pattern as in *C. a. gilcolladoi,* with differences in the three parameters between crop types (prevalence: *Χ*^*2*^ = 24.15, *df* = 2, *P* < 0.001; intensity: *Χ*^*2*^ = 30.45, *df* = 2, *P* < 0.001; abundance: *Χ*^*2*^ = 43.28, *df* = 2, *P* < 0.001) and months (prevalence: *Χ*^*2*^ = 26.82, *df* = 2, *P* < 0.001; intensity: *Χ*^*2*^ = 13.23, *df* = 2, *P* = 0.001; abundance: *Χ*^*2*^ = 34.53, *df* = 2, *P* < 0.001). Infestation with *N. fasciatus* was more frequent and severe during July, and lower levels of flea infestation were found in voles from alfalfas. In *A. sylvaticus*, *N. fasciatus* abundance did not differ between sexes, but varied between months (prevalence: *Χ*^*2*^ = 18.48, *df* = 2, *P* < 0.001; intensity: *Χ*^*2*^ = 5.92, *df* = 2, *P* = 0.052*; abundance: *Χ*^*2*^ = 18.14, *df* = 2, *P* < 0.001), with a higher infestation rate during July and a reduced intensity and abundance during November. The infestation with this flea varied between months in *M. spretus* although the effect was only marginally significant (prevalence: *Χ*^*2*^ = 5.36, *df* = 2, *P* = 0.069*; intensity: NA; abundance: *Χ*^*2*^ = 5.48, *df* = 2, *P* = 0.065*). The highest prevalence rate was found in July but the mean abundance dropped in March. In *M. spretus, N. fasciatus* was also more abundant on males than females (*Χ*^*2*^ = 2.74, *df* = 1, *P* = 0.0098*). The small sample size for this species did not allow modelling intensity variation.

Regarding *L. taschenbergi*, we found that this flea was more abundant on males than on females in *M. arvalis* (prevalence: *Χ*^*2*^ = 3.47, *df* = 1, *P* = 0.062*; intensity: *Χ*^*2*^ = 4.56, *df* = 1, *P* = 0.032; abundance: *Χ*^*2*^ = 4.50, *df* = 1, *P* = 0.034). In both mouse species, we found a significant effect of sex (*A. sylvaticus* prevalence: *Χ*^*2*^ = 16.68, *df* = 1, *P* < 0.001; intensity: *Χ*^*2*^ = 7.28, *df* = 1, *P* = 0.007; abundance: *Χ*^*2*^ = 16.69, *df* = 1, *P* < 0.001; *M. spretus* prevalence: *Χ*^*2*^ = 4.30, *df* = 1, *P* = 0.038; intensity: NA; abundance: *Χ*^*2*^ = 4.80, *df* = 1, *P* = 0.029) and month (*A. sylvaticus* prevalence: *Χ*^*2*^ = 54.53, *df* = 2, *P* < 0.001; intensity: *Χ*^*2*^ = 56.34, *df* = 2, *P* < 0.001; abundance: *Χ*^*2*^ = 97.93, *df* = 2, *P* < 0.001; *M. spretus*: prevalence: *Χ*^*2*^ = 7.15, *df* = 2, *P* = 0.028; intensity: NA; abundance: *Χ*^*2*^ = 9.01, *df* = 2, *P* = 0.011). The infestation was more prevalent and severe in July and among males than females in both host species. Furthermore, crop type explained variations in intensity and abundance in *A. sylvaticus* (intensity: *Χ*^*2*^ = 7.86, *df* = 2, *P* = 0.020; abundance: *Χ*^*2*^ = 6.15, *df* = 2, *P* < 0.046), with a greater intensity in hosts from cereal fields compared with fallows.

### Co-infections

The majority of hosts were infested with one or two flea species (63.2% and 34.5%, respectively). Few hosts (2.3%) harboured three flea species (Table 3). Higher co-infection rates were found in *M. arvalis* (38.8%) and *A. sylvaticus* (34.6%) than in *M. spretus* (13.3%) and *C. russula* (16.7%). Co-infection patterns with two flea species were diverse among hosts. Associations composed of *C. a. gilcolladoi-N. fasciatus* were most commonly found in *M. arvalis* (90.3%), and very few co-infections with *L. taschenbergi*. In fact, this flea was collected alone in almost 90% of the cases. *L. taschenbergi*-*N. fasciatus* associations prevailed in the mouse hosts (*A. sylvaticus =* 45.9%; *M. spretus =* 66.7%), although this predominance in *A. sylvaticus* was similar to the other co-infections (*C. a. gilcolladoi–N. fasciatus*: 30.3% and *C. a. gilcolladoi–L. taschenbergi*: 23.9%; Fig. 1). These results were in agreement with the Fager index values obtained for pairs of flea species on these hosts (Table 3).

**Table 3:** Flea co-infection rates on the main hosts and Fager index for the most common flea associations

Host	Flea species per host			Fager index		
	Prevalence% [*n* host]			[*n* host]		
	1	2	3	CAG-LT	CAG-NF	LT-NF
*M. arvalis*	61.2 [534]	37.2 [324]	1.6 [14]	0.046 [13]	0.538 [318]	0.063 [21]
Male	64.1 [270]	34.0 [143]	1.9 [8]	0.060 [8]	0.498 [139]	0.098 [16]
Female	58.5 [264]	40.1 [181]	1.3 [6]	0.029 [5]	0.574 [179]	0.029 [5]
*A. sylvaticus*	65.1 [155]	28.7 [68]	5.9 [14]	0.242 [26]	0.346 [33]	0.391 [50]
Male	60.2 [97]	31.7 [51]	8.1 [13]	0.273 [22]	0.424 [28]	0.442 [40]
Female	76.3 [58]	22.4 [17]	1.3 [1]	0.148 [4]	0.169 [5]	0.267 [10]
*M. spretus*	86.7 [39]	13.3 [6]	0	0.069 [1]	0.063 [1]	0.205 [4]
Male	83.3 [30]	16.7 [6]	0	0.083 [1]	0.077 [1]	0.250 [4]
Female	100.0 [9]	0	0	0	0	0
*C. russula*	83.3 [5]	16.7 [1]	0	0	0.286 [1]	0
Male	75.0 [3]	25.0 [1]	0	0	0.400 [1]	0
Female	100.0 [2]	0	0	0	0	0

1, one flea species; 2, two co-occurrence flea species; 3, three co-occurrence flea species. For flea species abbreviation (CAG, LT, NF) see Fig. 1

Co-infection rates did not differ between sexes in *M. arvalis* (*Χ*^*2*^ = 2.87, *df* = 1, *P* = 0.090) or in *M. spretus* (*G* = 2.90, *df* = 1, *P* = 0.886), but did in *A. sylvaticus* (*Χ*^*2*^ = 5.89, *df* = 1, *P* = 0.015), with fewer co-infections in female than male hosts. Considering hosts infested with two or three species, we found no differences between sexes (*M. arvalis*: *Χ*^*2*^ = 2.67, *df* = 1, *P* = 0.102; *A. sylvaticus*: *G* = 2.63, *df* = 1, *P* = 0.105). In terms of co-infection assemblies, male and female wood mice presented similar values of co-infection for all flea pairs (*G* = 0.87, *df* = 3, *P* = 0.649). In voles, however, we found a male-biased *N. fasciatus*-*L. taschenbergi* co-infection, which occurred more frequently in male than in female hosts (*Χ*^*2*^ = 7.54, *df* = 1, *P* = 0.006).

## Discussion

The flea community parasitizing the small mammal guild studied here was mainly (99.4%) composed by *N. fasciatus*, *C. a. gilcolladoi* and *L. taschenbergi* and showed species-specificity and marked aggregation patterns. We found strong sex-biased differences in the mouse hosts, lower flea infestation in voles captured from alfalfas, and seasonal variations differing between host and flea species. Interspecific co-infections were frequent, with up to three different flea species in some hosts.

### Flea community

The northern rat flea (*N. fasciatus*) was the most common and most abundant flea species in the studied small mammal community, parasitizing both rodents and insectivores. This flea mainly parasitizes rodents but can facultatively infest a wide range of mammalian hosts [48], which can explain their overall high abundance and prevalence rates. A pattern of generalist fleas reaching heavier infestations has also been reported in other systems [49]. *Ctenophthalmus* fleas are a generalist group that diverges in their distribution range owing to geographical specificity [50]. Although all *Ctenophthalmus* species identified here are endemic to the Western Mediterranean area, *C. a. gilcolladoi* is the typical flea of the open habitats of this region [50, 51]. *Leptopsylla taschenbergi* was the most abundant flea found on *A. sylvaticus* and *M. spretus*, and showed a low infestation rate on common voles, which is consistent with its well-known mouse-specificity [51].

Our results show the typical aggregation pattern of fleas on rodents found elsewhere [52]. Poulin D-index values for the most abundant fleas (*N. fasciatus *and *C. a. gilcolladoi*) were lower in *M. arvalis* than in mice. Behavioural traits could explain these differences [19], since the social behaviour of common voles could facilitate the switching of fleas between hosts, reducing flea aggregation. Regarding variance-to-mean ratios, we found differences between *N. fasciatus* and *C. a. gilcolladoi* despite similar abundances. The host-generalist strategy of *N. fasciatus* [5] could explain the reduced aggregation level. Generalist parasites with broad habitat requirements, like *N. fasciatus*, would have access to more hosts, having more resources available and reducing intraspecific competition. Another important aspect to consider is the local asynchrony in abundance fluctuations of the rodent species (summer peak in voles versus autumn peaks in mice; [13, 53]). Thereby, many potential hosts are accessible to generalist fleas during those high density periods throughout the year, potentially causing a dilution effect for *N. fasciatus* and lower its aggregation levels. Conversely, highly specialised fleas (such as *L. taschenbergi*) would tend to parasitize only the fewer suitable hosts that are available (i.e. *A. sylvaticus* and *M. spretus*).

### Variations of flea parasitological parameter variation according to season, crop type and host sex

*Microtus arvalis* harboured the highest flea burden and infestation rate, followed by *A. sylvaticus*. Larger body size and greater complexity of the burrow system can lead to heavier flea infestations [54], which could explain the lower infestation rates in *M. spretus* and *C. russula* (although the sample size of the latter was too small to draw a solid conclusion). Moreover, the fossorial life of *M. arvalis* could increase the probability of getting infested by naïve fleas because burrows are used by preimaginal stages to develop until their first blood feeding [5].

We found seasonal variations in the main flea species whatever the rodent host. In general terms, prevalence models could be easily understood from a flea phenology point of view, since maximum values occurred during the season with the greatest flea activity: *C. a. gilcolladoi* showed higher presence in March/November, with a significant drop in July, and vice versa for *N. fasciatus* and *L. taschenbergi*, which is in accordance with previous studies [5, 48, 51]. At a global scale, *N. fasciatus* has no marked seasonality in Atlantic conditions [5]. However, *N. fasciatus* eggs require at least 23 °C during 4 days or a peak of 30 °C for 3 h to hatch [55, 56]. The extreme cold conditions during the autumn-winter months in the study region (including November and March) may lead this species to shorten its reproductive period or modify its activity pattern, recovering in spring as the weather conditions become more suitable.

Seasonality in flea patterns is common as are variations owing to local climatic conditions and host traits [18, 22–24]. Assuming the same phenology in individuals belonging to a certain species living under the same climatic conditions, differences in infestation parameters can be explained by host characteristics. The patterns obtained in this study also seemed to be influenced by particular hosts traits. Prevalence models followed the same general rule according to flea phenology, but many seasonal peculiarities arose considering variations in intensity regarding one host that did not show the others. Overall, maximum infestation rate occurred during summer, when small mammal species are more active and reproduce [35], potentially increasing the exposure of hosts through contacts with other infested animals [19]. However, we found that maximum infestation rate and maximum intensity occurred simultaneously during this favourable season in mice, but not in voles. The low flea abundance in voles during this favourable season for transmission, despite a high percentage of infected hosts, could be explained by a dilution effect owing to the increase in the population density of voles compared to colder months (see [13] for more details about host density dynamics). Similarly, the intensity of *N. fasciatus* also differed between voles and mice during July. Since the intensity and prevalence of voles increased significantly during the summer, the reproductive period of this flea [51], infestation burden remained low in mice until March. If burrows and mice themselves were as suitable as voles, no differences in intensity should exist between the two type of rodents. Nevertheless, intensity remained low in mice during the most suitable season for *N. fasciatus* reproduction. A possible reason could be that wood mouse nests or their own body are less suitable for this flea compared with voles. This type of differences at the host species level has been described in other small mammal communities [57]. Therefore, the maximum intensity of *N. fasciatus* in mice is reached at minimum mouse population abundance (after the winter mortality and before the spring reproductive recruitment) [13], which may facilitate the flea aggregation in the surviving animals. Furthermore, *A. sylvaticus* have a more individualistic behaviour and spend more time inside the burrows during the cold months [33], which could reduce the probability of flea transmission to other hosts, preventing a decrease in flea intensity [19].

A similar pattern may be occurring for *C. a. gilcolladoi* on wood mice in March when prevalence patterns differed from those on voles. A noticeable seasonality was found in the latter, following the typical flea phenology with peaks during temperate months [5, 51]. We found lower infestation values and no differences in prevalence throughout the year in mice. These results suggest that *C. a. gilcolladoi* may prefer infesting common voles rather than mice in this ecosystem, although no investigation has been done yet to determine the relationship between *C. a. gilcolladoi* and their hosts in this system. This possible preference was not expected since *M. arvalis* is a host species that colonized the study region during the 1970s, invading from mountain habitats characterized by Atlantic weather conditions [10]. *Ctenophthalmus apertus* is endemic to Mediterranean open habitats and absent from Atlantic climatic areas [50]; thus, it does not occur within the original distribution range of *M. arvalis*. A host shift could have possibly occurred if this new host species offered better conditions than other hosts [57]. The colonization by a new species, the common vole, may therefore have altered the host-parasite system. If either of them acts as a reservoir or vector of any pathogen, the consequences and implications of this alteration could be unexpected [58, 59]. Future investigation could elucidate the consequences of such shifts in terms of pathogen transmission risk.

We also found differences in flea parameters depending on the habitats used by hosts. Common voles inhabiting alfalfas had lower flea prevalence and abundance than those from other crop types. *Leptopsylla taschenbergi* infestation in voles also appeared associated with cereal habitats. Wood mice inhabiting cereals had heavier flea burdens compared with other habitats. Some authors have linked animal condition and abundance with the quality and quantity of food supply [60]. The most favourable crop type for voles is alfalfa, where they reach the highest densities and better body condition [60]. The wood mouse has a strong preference for woody habitats [61], avoiding habitats without shrub [62] and with insufficient cover to protect them from predators [63] such as cereals (especially recently sown crops in November and stubbles in July). Animals inhabiting sub-optimal habitats may have greater flea infestation because they are in worse condition, favouring the egg production and survival of flea larvae [64].

Lastly, we found sexual differences in flea infestation in both mice species, with greater prevalence and severity in male than in female hosts. Such patterns were not found in *M. arvalis*, except with the less frequent flea species, *L. taschenbergi*. This male-biased difference is usually linked to sexual size dimorphism, immunosuppression by sexual hormones, or behavioural differences that facilitates flea encounter and horizontal transmission [65]. Such patterns have been already reported in several mice species [22, 66, 67]. However, the absence of sex bias in our voles suggests that differences at the host scale may cause these dissimilarities. This may be due to the colonial lifestyle [68] and the aggressive behaviour of female voles [69] that could increase the horizontal transmission among females, balancing the flea burden between both sexes. Male voles are more mobile than females [68] and also more active [70] and therefore more prone to flea encounters [5], potentially explaining why the less frequent flea species (*L. taschenbergi*) occurred more often in male than in female voles.

### Co-infections and implications

In the Mediterranean agricultural landscapes studied here, two dominant flea species (*N. fasciatus* and *C. a. gilcolladoi*) were shared by 40% of the hosts of the small mammal guild. Flea co-occurrence is common in small mammals [71] and maybe due to apparent facilitation via suppression of the host immune system [72]. It is known that different fleas can be infected by the same pathogen [30], while several pathogens can be harboured by a shared host [7]. Since fleas are important vectors for many pathogens [1], coinfections could have important consequences in terms of disease dispersal and zoonotic transmission risk [73, 74], increasing the circulation of pathogens through a shared host population. Moreover, generalist and abundant fleas can infest animals living next to people and, eventually, bite humans. The generalist flea *N. fasciatus* was found in the most abundant co-infections of the four small mammals, with an apparently high tolerance to cohabit with other flea species. In fact, we identified this flea and *C. a. gilcolladoi* (the two more abundant fleas) in 82.7% of all the co-infections analysed. Notably, previous work [30] detected *Bartonella* spp. prevalences of 65% in *N. fasciatus* collected from our focal common vole population and of 33% in *C. a. gilcolladoi*. Additional investigation should therefore be carried out to determine the potential roles of these two fleas in the transmission of bartonelloses. Moreover, *N. fasciatus* is a typical parasite of *Rattus* spp. [1], and both fleas are known to parasitize *Mus domesticus* in Spain [75]. *Rattus* and *Mus* spp. commensal rodents are widespread in rural areas, especially linked to the presence of domestic livestock, and they can be infected by *Bartonella* sp. [76, 77]. Density can influence the flea-host system [20, 21], and fluctuations in wild rodent densities can facilitate the encounters with commensal rodents, fleas and humans, increasing the possibility of disease transmission in the rural population inhabiting this agricultural area. The other flea species (other *Ctenophthalmus* spp. and *R. beillardae*) were more rarely found. Therefore, their possible role in the circulation cycle of zoonoses among this small mammal host guild would probably be less relevant.

## Conclusion

Six different flea species parasitized the studied small mammal guild inhabiting continental Mediterranean farmland, although *C. a. gilcolladoi* and the generalist *N. fasciatus* were found to dominate the flea community. Co-infections with both flea species, which often harbour zoonotic pathogens, frequently occurred within the focal host guild. The role that flea species might play in zoonotic transmission should be elucidated, considering also seasonal patterns and sex-biased differences. Moreover, the most abundant host (i.e. *M. arvalis*) is a recent colonizer and, unlike other small mammal hosts, is characterized by large fluctuations in abundance. The consequences of fluctuating *M. arvalis* abundances for the transmission cycles of flea-vectored diseases should also be investigated.

## Supplementary information

**Additional file 1: Figure S1.** Flea cumulative frequencies in the main small mammal host species. CAA, *Ctenophthalmus apertus apertus*; CAG, *Ctenophthalmus apertus gilcolladoi*; CB, *Ctenophthalmus baeticus*; NF, *Nosopsyllus fasciatus*; LT, *Leptopsylla taschenbergi*; RB, *Rhadinopsylla beillardae*. * Sample size too small (*n* = 1).

## Data Availability

The datasets used and/or analysed during the current study are available from the principal investigator JJ Luque-Larena (j.luque@agro.uva.es) on reasonable request.
