# Supplementary figures and images for: Patterns of flea infestation in rodents and insectivores from intensified agro-ecosystems, Northwest Spain
Source: Parasit Vectors. 2021 Jan 6;14:16. doi: 10.1186/s13071-020-04492-6 (PMC7789319; doi:10.1186/s13071-020-04492-6)

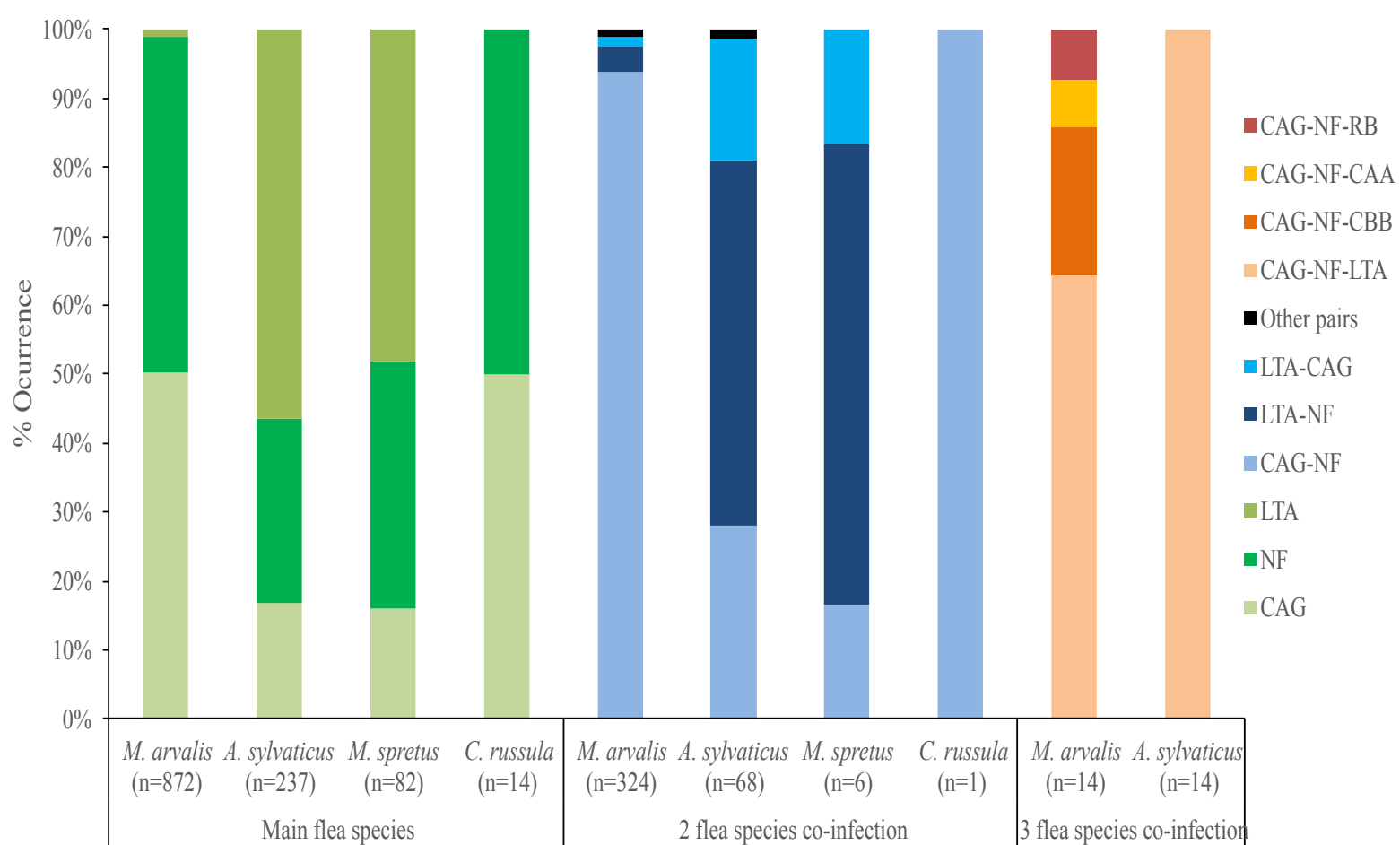

Supplement: Supplementary file 1 — Additional file 1: Figure S1. Flea cumulative frequencies in the main small mammal host species. CAA, Ctenophthalmus apertus apertus; CAG, Ctenophthalmus apertus gilcolladoi; CB, Ctenophthalmus baeticus; NF, Nosopsyllus fasciatus; LT, Leptopsylla taschenbergi; RB, Rhadinopsylla beillardae. * Sample size too small (n = 1). [file 13071_2020_4492_MOESM1_ESM.pdf]
